# Supplementary material for: The spatial organization of intra-tumour heterogeneity and evolutionary trajectories of metastases in hepatocellular carcinoma
Source: Nat Commun. 2017 Feb 27;8:4565. doi: 10.1038/ncomms14565 (PMC5333358; doi:10.1038/ncomms14565)
Supplement: Supplementary Information — Supplementary Figures, Supplementary Tables, Supplementary Notes and Supplementary References [file ncomms14565-s1.pdf]

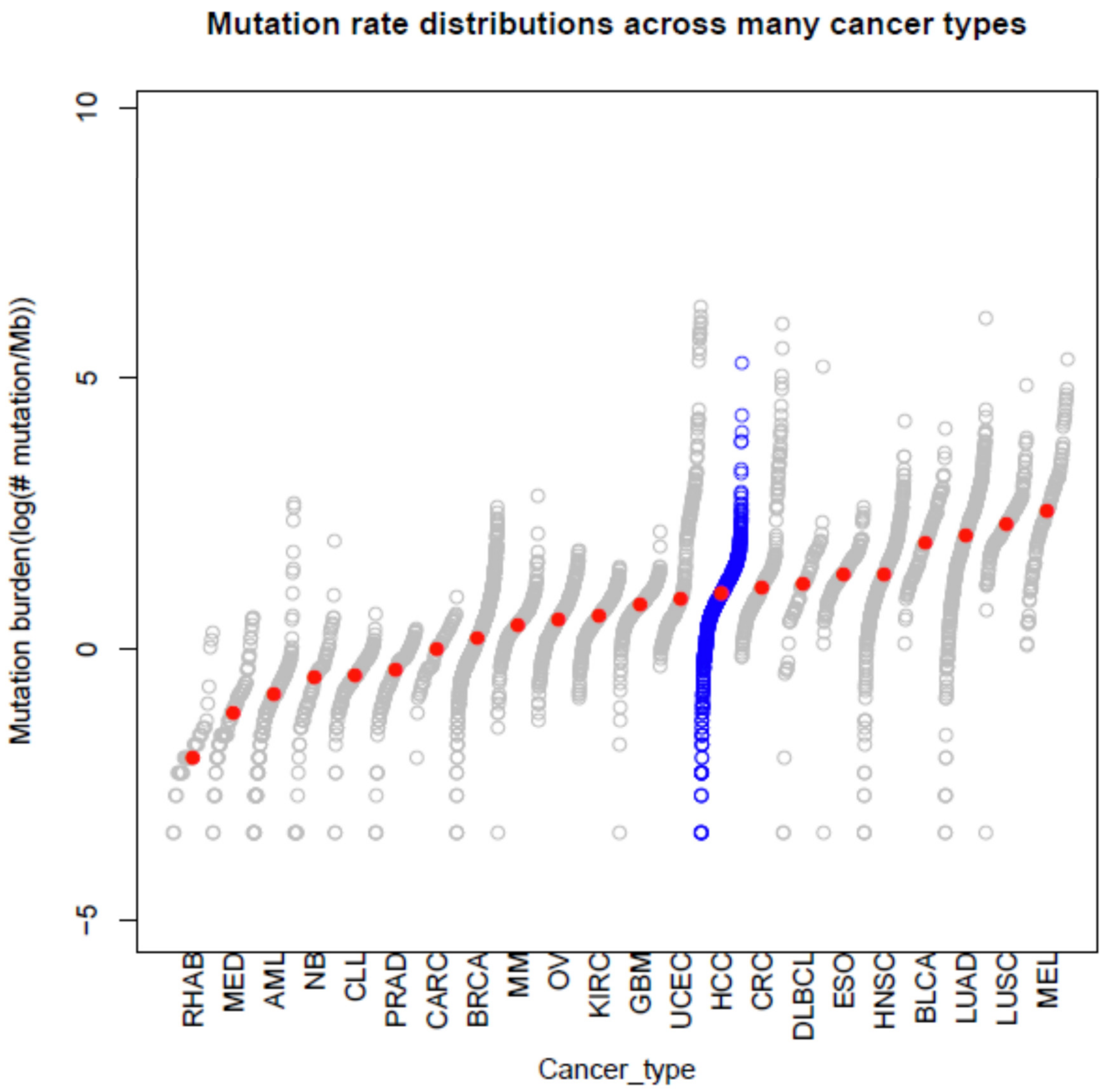


**Supplementary Figure 1. Mutation rate distributions across many cancer types**

Data other than HCC were extracted from Lawrence et al published at http://www.tumorportal.org/. Red dots are showing the median rate for that cancer type. AML: Acute Myeloid Leukemia, BLCA: Bladder, BRCA: Breast, CARC: Carcinoid, CLL: Chronic lymphocytic leukemia, CRC: Colorectal, DLBCL: Diffuse large B-cell lymphoma, ESO: Esophageal adenocarcinoma, GBM: Glioblastoma multiforme, HNSC: Head and Neck, KIRC: Kidney clear cell, LUAD: Lung adenocarcinoma, LUSC: Lung squamous cell carcinoma, MED: Medulloblastoma, MEL: Melanoma, MM: Multiple myeloma, NB: Neuroblastoma, OV: Ovarian, PRAD: Prostate, RHAB: Rhabdoid tumor, UCEC: Endometrial, HCC: Hepatocellular Carcinoma.

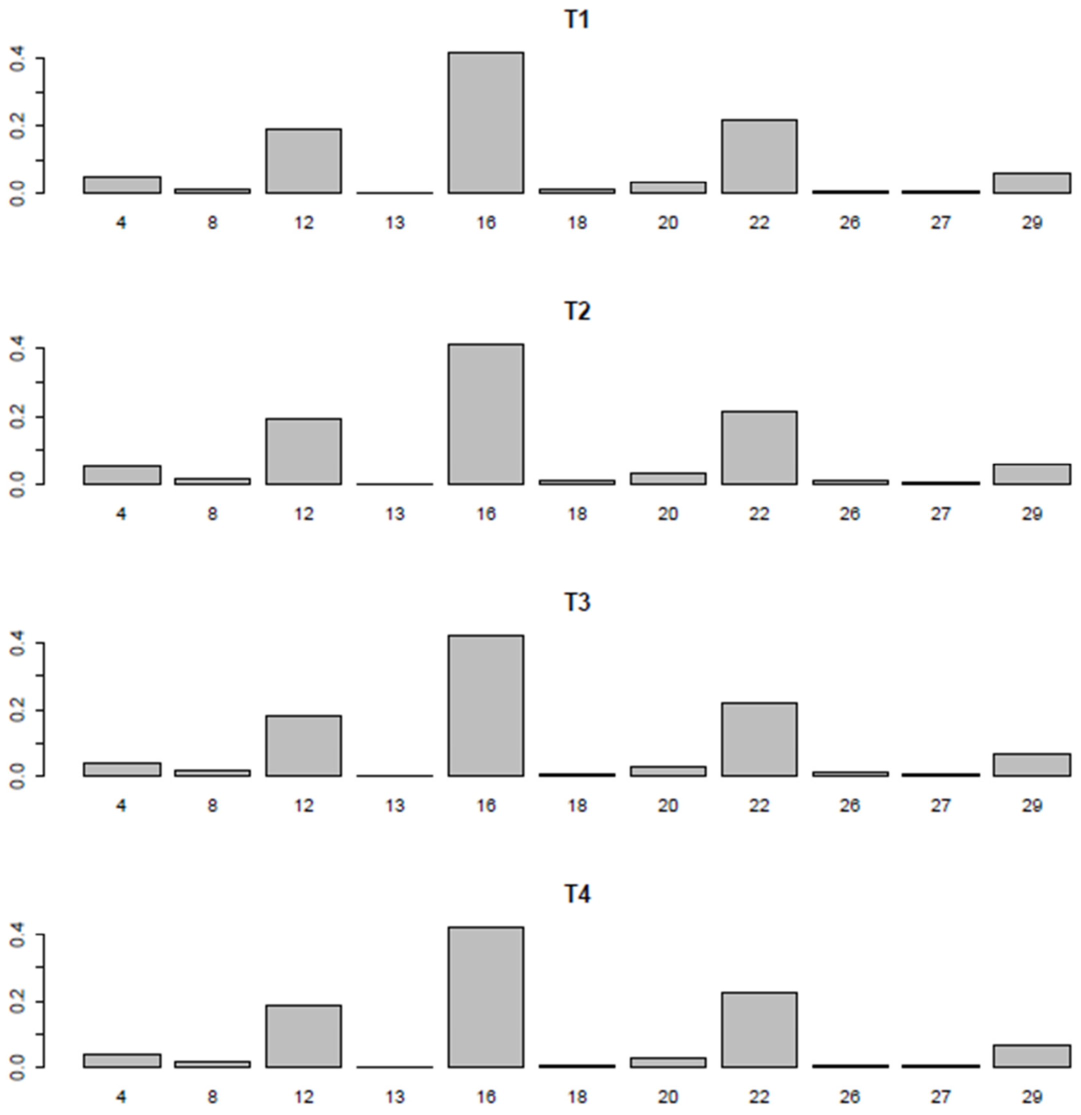


**Supplementary Figure 2. The mutation signatures in patient 9.**

The X axis is the name of the mutational signatures (1-30). The Y axis is the proportion contributed by that signature. Using 30 existing mutation signatures from the COSMIC database, we projected the mutations into possible combinations of each mutation signature for each sector. Several mutation signatures (including the AA signature/mutation Signature 22) that are strongly contributing to the patient mutational profile (mutation signature of less than 0.0001 proportions are not plotted).

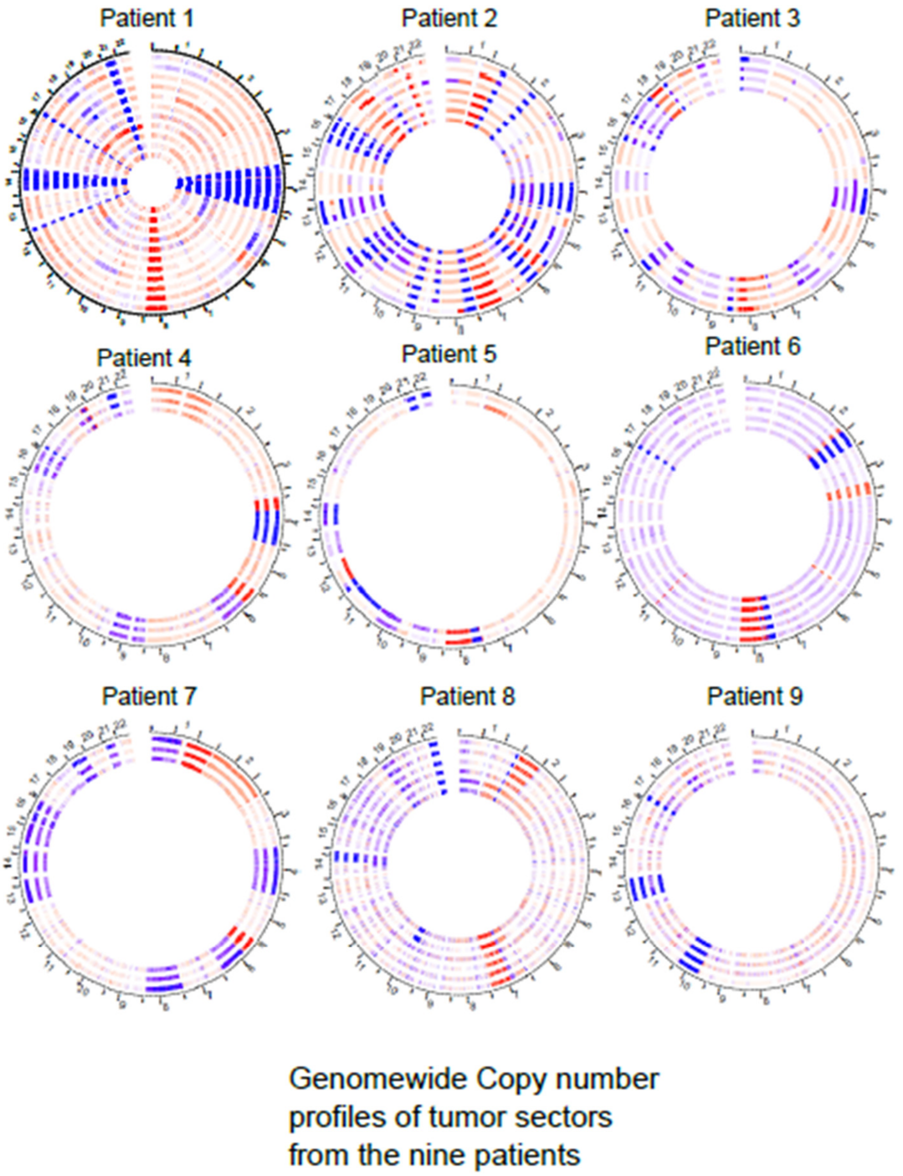


**Supplementary Figure 3. Genome wide copy number across nine patient samples (Circos plot)** Tumors are orders from inner circle to outer circle (i.e. T1 is the most inner circle). Red represents amplifications and blue represents deletions. The chromosomes are ordered around the circle.

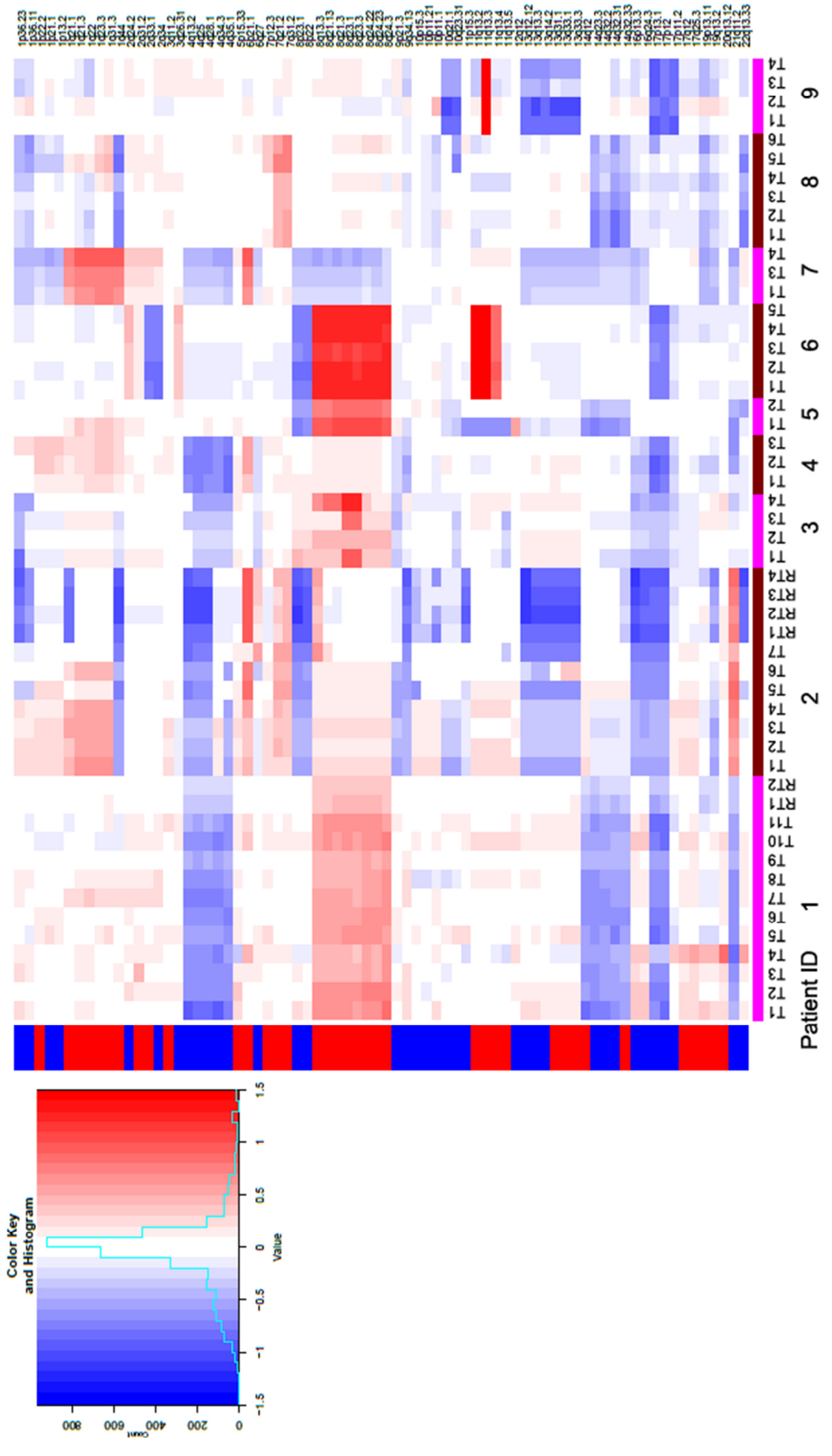


**Supplementary Figure 4. CNV profile at the GISTIC cytoband level**

Red represents amplifications and blue represents deletions. The side bar represents the results from the GISITC results from a large number of patients. The scale of colors is show as the side legend.

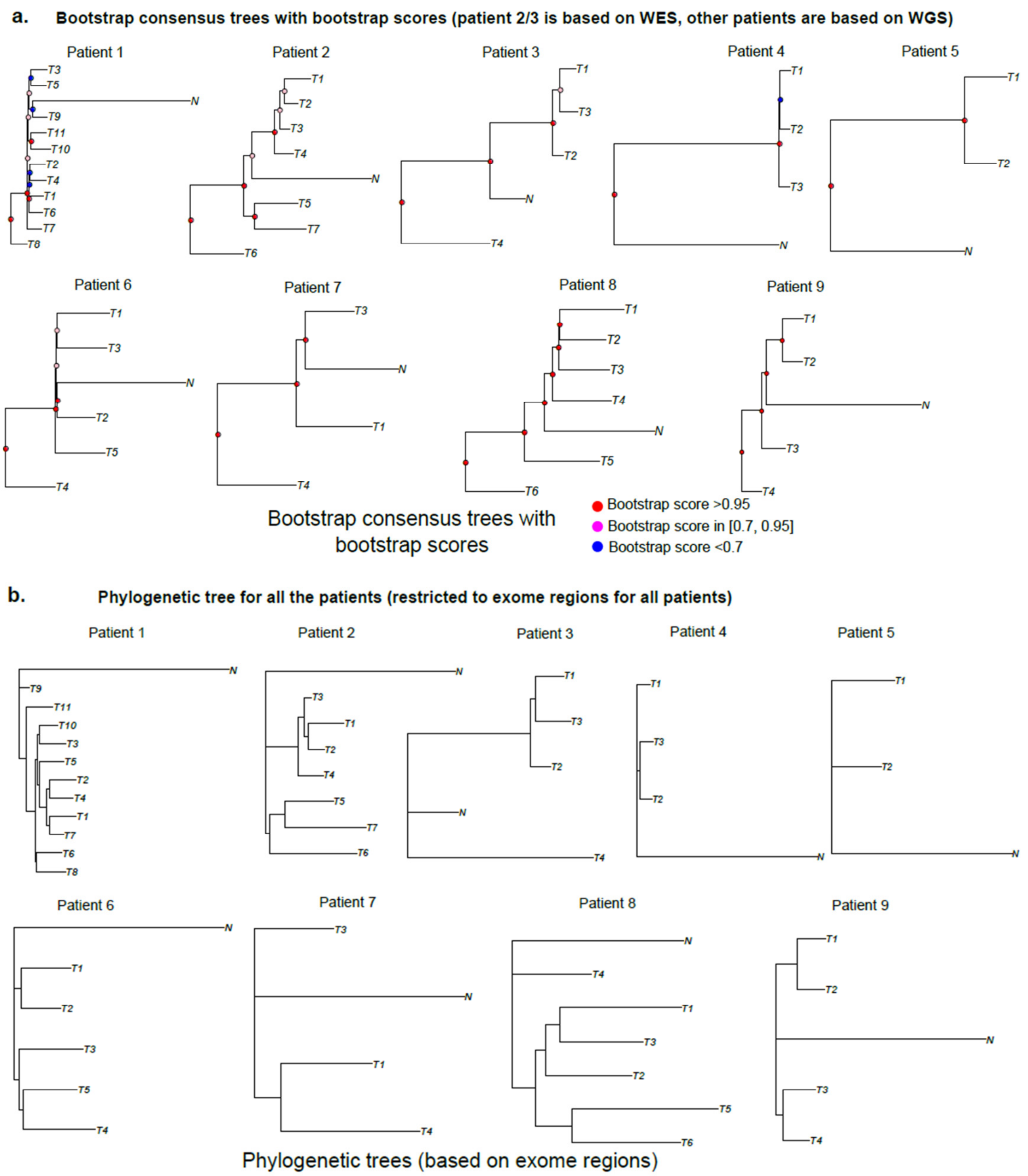


**Supplementary Figure 5. Consensus tree and the bootstrap score**

a) Bootstrap consensus tree of all patients using WGS (patient 1/4/5/6/7/8/9) as well as WES (patient 2/3). Each internal node is marked by their bootstrap score (red/pink/blue marks nodes with different statistical confidence). Bootstrap scores >0.7 is often regarded as high confidence nodes in statistical phylogenetics. b) Phylogenetic trees constructed using the exome part of the genome (patient 2/3 are the same as panel a).

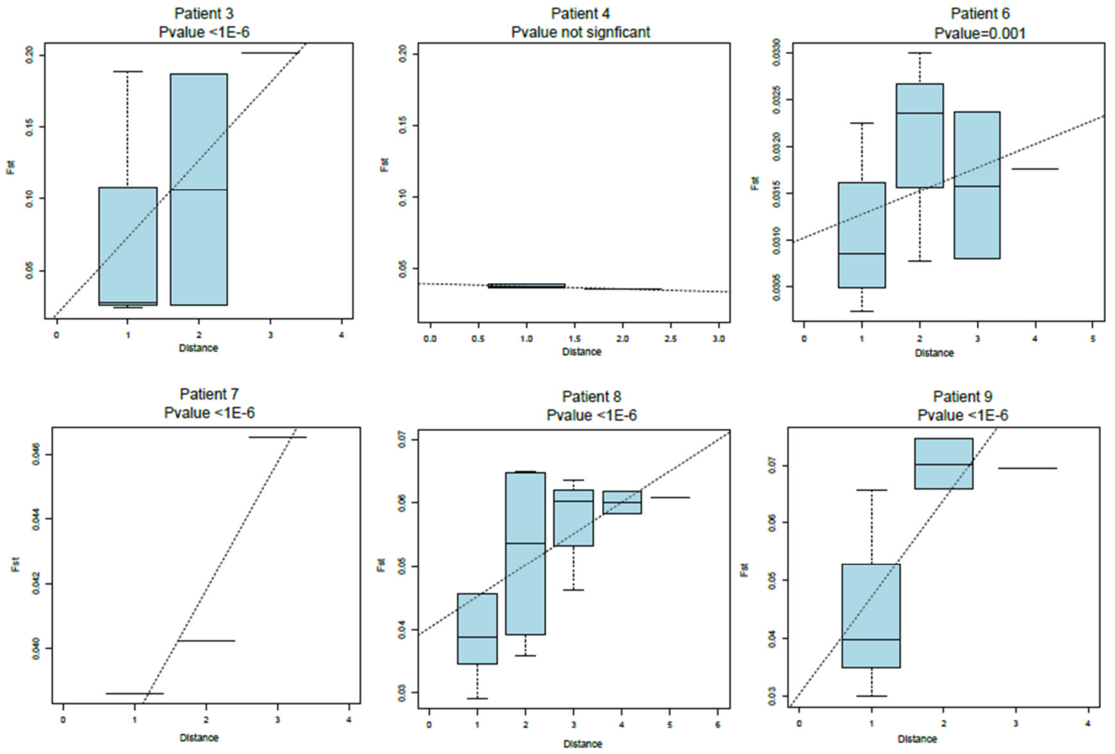


**Supplementary Figure 6. Isolation by distance relationship between tumor sectors for patient 3-9 (except patient 5)** The x-axis is the physical distance between sectors and the y-axis is the genetic differentiation (Fst) between the samples. Fst values from all sector pairs with the same physical distance were used to draw boxplots at each distance value. The regression line and the pvalue are derived from the linear regression model (see Methods). Patient 5 is dropped from the linear regression because we only have two sectors for this patient. Patient 1 and 2 are in the maintext (Figure 3b). Pvalues are based on the linear model fitting between Fst and physical distances (Methods).

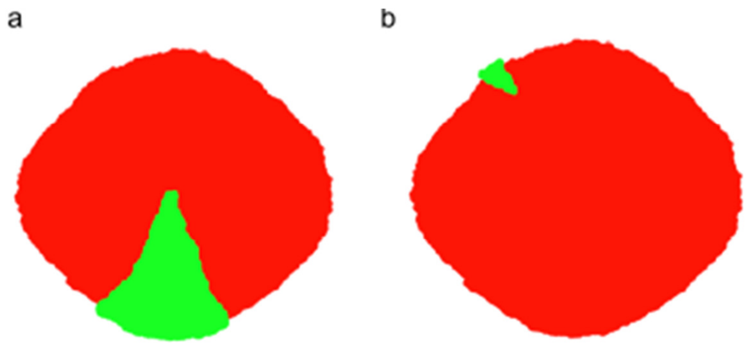

**Supplementary Figure 7. the geographic locations of the different mutations in the computer simulation.** Two different mutations are shown as examples. a) Mutations which arise early tend to be distributed along a wider geographic location. Cells carrying the wild type allele are plotted as red dots and cells carrying the mutation are shown as green dots. b) Mutations which arise late will be restricted to a smaller region.

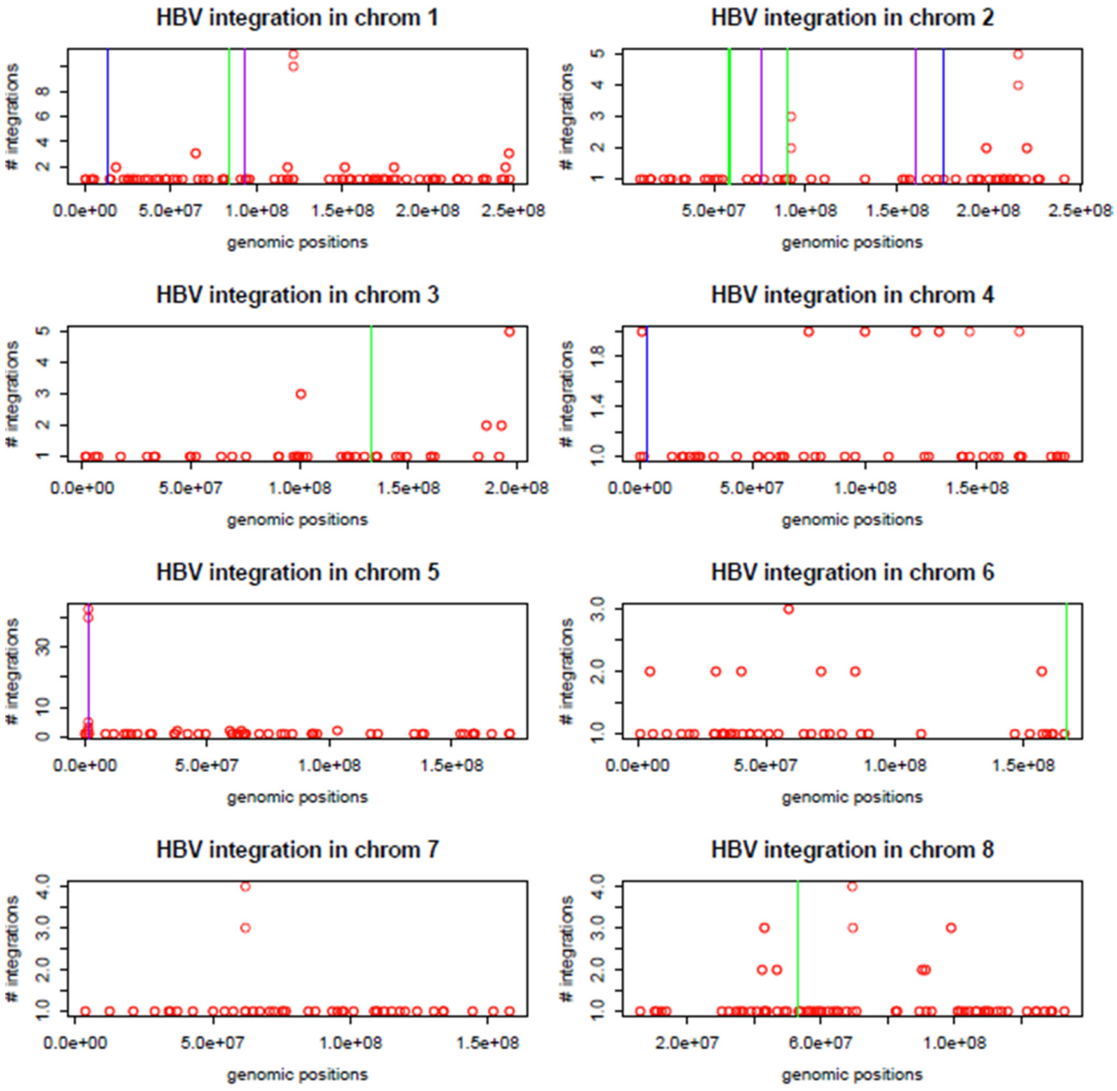
HBV integration in chrom 1
# integrations
genomic positions
HBV integration in chrom 2
# integrations
genomic positions
HBV integration in chrom 3
# integrations
genomic positions
HBV integration in chrom 4
# integrations
genomic positions
HBV integration in chrom 5
# integrations
genomic positions
HBV integration in chrom 6
# integrations
genomic positions
HBV integration in chrom 7
# integrations
genomic positions
HBV integration in chrom 8
# integrations
genomic positions

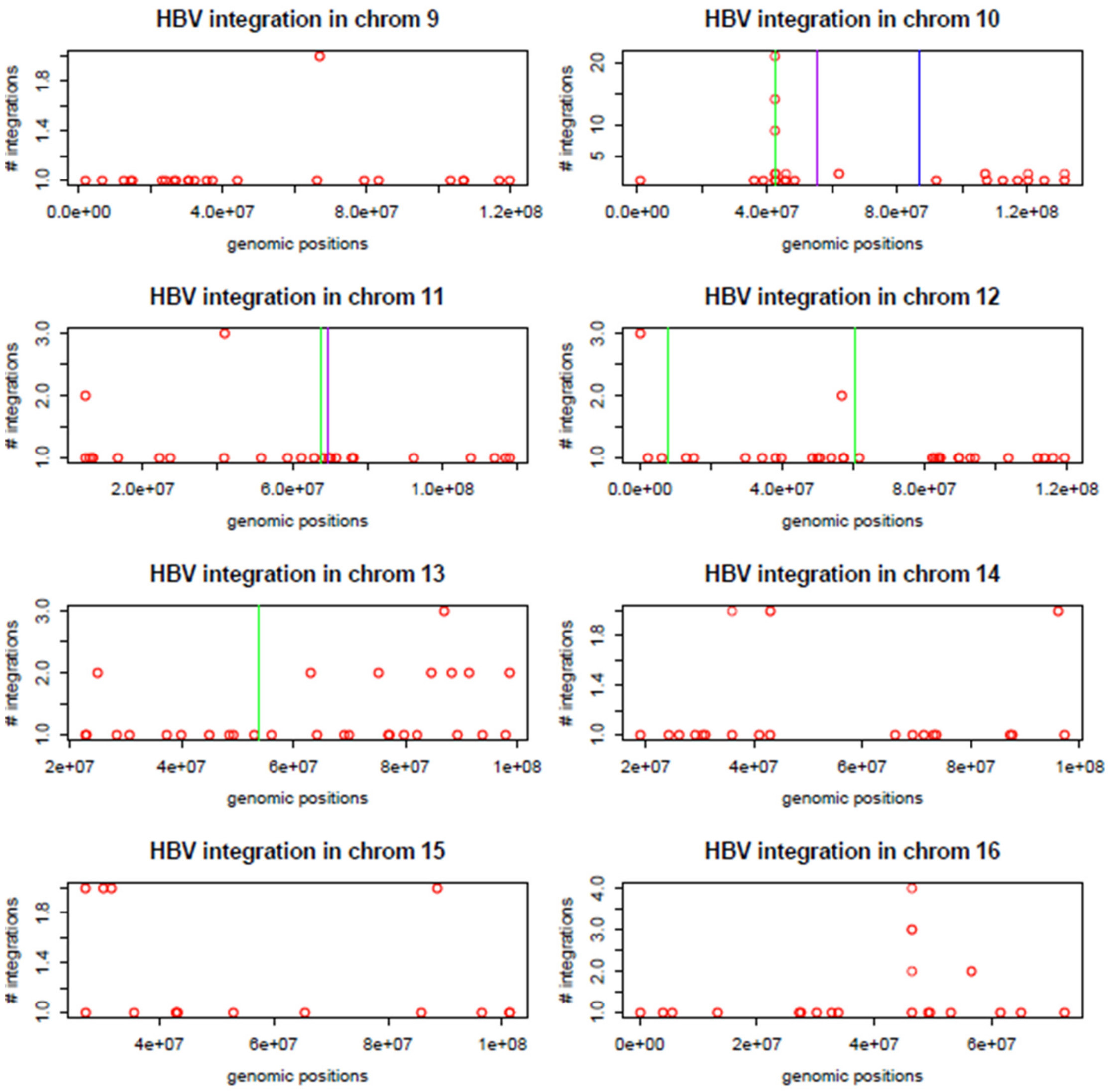
HBV integration in chrom 9
# integrations
1.0
1.4
1.8
0.0e+00
4.0e+07
8.0e+07
1.2e+08
genomic positions
HBV integration in chrom 10
# integrations
5
10
20
0.0e+00
4.0e+07
8.0e+07
1.2e+08
genomic positions
HBV integration in chrom 11
# integrations
1.0
2.0
3.0
2.0e+07
6.0e+07
1.0e+08
genomic positions
HBV integration in chrom 12
# integrations
1.0
2.0
3.0
0.0e+00
4.0e+07
8.0e+07
1.2e+08
genomic positions
HBV integration in chrom 13
# integrations
1.0
2.0
3.0
2e+07
4e+07
6e+07
8e+07
1e+08
genomic positions
HBV integration in chrom 14
# integrations
1.0
1.4
1.8
2e+07
4e+07
6e+07
8e+07
1e+08
genomic positions
HBV integration in chrom 15
# integrations
1.0
1.4
1.8
4e+07
6e+07
8e+07
1e+08
genomic positions
HBV integration in chrom 16
# integrations
1.0
2.0
3.0
4.0
0e+00
2e+07
4e+07
6e+07
genomic positions

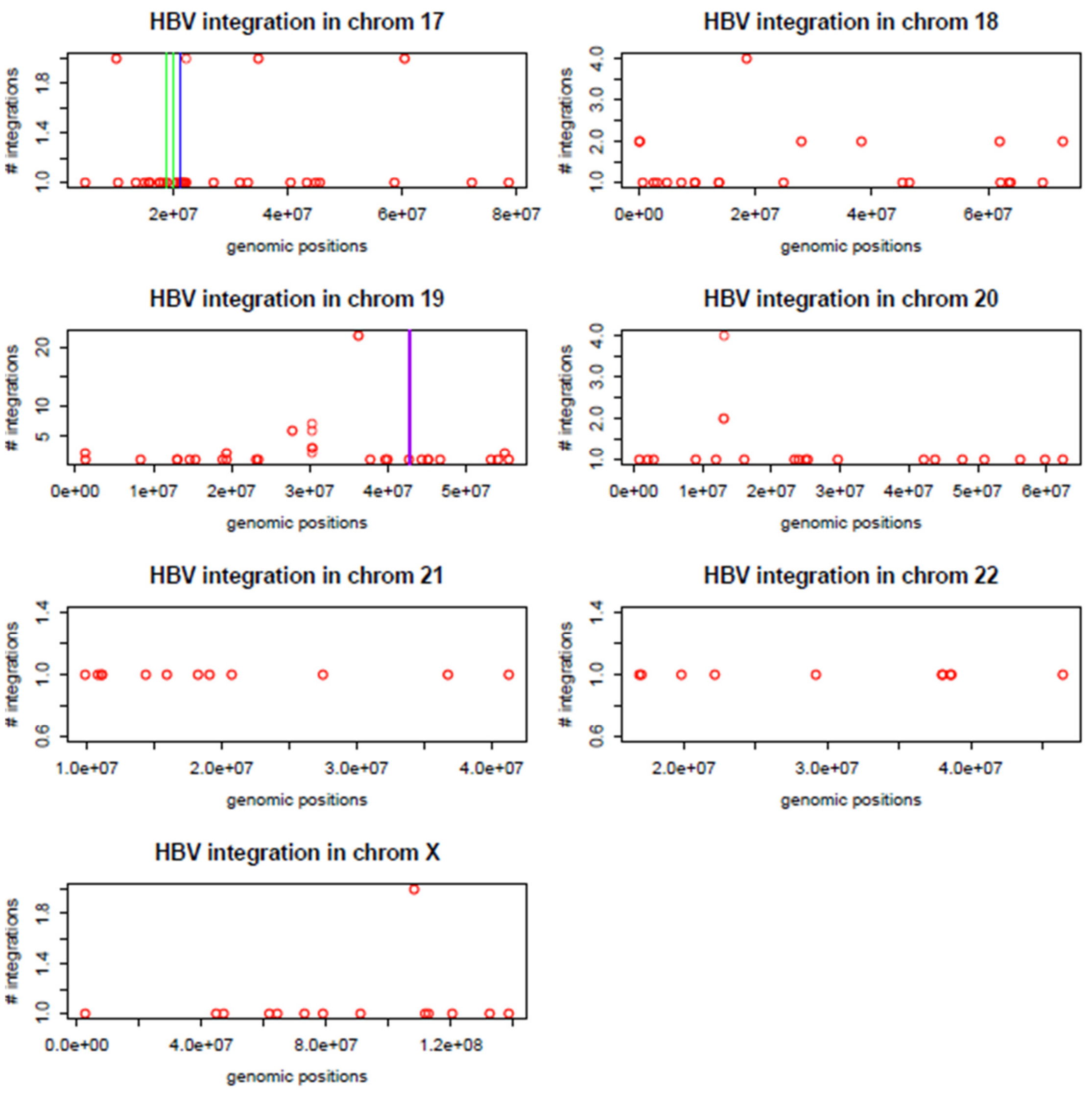


**Supplementary Figure 8. Sliding window of integration hotspots and the position of the integrations found in our datasets** The red dots indicate the results from the sliding window analysis of the public integrations (1027 integrations). The window is set to be 20kb with a step size of 10kb. The number of integrations is shown in the y-axis. The blue vertical lines mark the integrations found in the first patient and the green vertical lines mark the integrations found in the sixth patient and purple vertical lines mark the integrations from patient eight.

**Supplementary Table 1. Patient information for this study**

| Patient | Age | Gender | Ethnicity | Viral | TNM | Cirrhosis (stage) |
|---|---|---|---|---|---|---|
| 1 | 44 | M | Malay | HBV | T2N0M0 | Cirrhosis(4) |
| 2 | 73 | M | Indonesian Chinese | Nonviral | T2N0M0 | No |
| 3 | 64 | F | Indonesian Chinese | HBV | T2N0M0 | Cirrhosis(4) |
| 4 | 68 | F | Myanmar | HCV | T2N0M0 | Cirrhosis(4) |
| 5 | 62 | M | Cambodia | HCV | T2N0M0 | Cirrhosis(4), 50% Steatosis |
| 6 | 57 | M | Indonesian | HBV | T1N0M0 | Cirrhosis(4) |
| 7 | 30 | M | Chinese | HBV | T1N0M0 | Cirrhosis(4) |
| 8 | 66 | M | Chinese | HBV | T1N0M0 | Cirrhosis(4) |
| 9 | 59 | M | Burmese | HCV | T1N0M0 | Cirrhosis(4) |

**Supplementary Table 2. Sequence throughput for this study**

| Patient | Library | Sector | Type | Coverage |
|---|---|---|---|---|
| Patient 1 | WHL003 | N | WGS | 37.16 |
| Patient 1 | WHL004 | T2 | WGS | 38.65 |
| Patient 1 | WHL005 | T1 | WGS | 35.98 |
| Patient 1 | WHL006 | T3 | WGS | 34.26 |
| Patient 1 | WHL007 | T4 | WGS | 34.76 |
| Patient 1 | WHL008 | T5 | WGS | 35.28 |
| Patient 1 | WHL009 | T6 | WGS | 36.41 |
| Patient 1 | WHL010 | T7 | WGS | 34.58 |
| Patient 1 | WHL011 | T8 | WGS | 34.97 |
| Patient 1 | WHL012 | T9 | WGS | 35.39 |
| Patient 1 | WHL013 | T10 | WGS | 35.35 |

| | | | | |
|---|---|---|---|---|
| Patient 1 | WHL014 | T11 | WGS | 35.87 |
| Patient 1 | WHL380 | RT1 | WGS | 39.61 |
| Patient 1 | WHL381 | RT2 | WGS | 36.32 |
| Patient 2 | CHL450 | N | WES | 100.58 |
| Patient 2 | CHL451 | T2 | WES | 86.61 |
| Patient 2 | CHL452 | T3 | WES | 53.46 |
| Patient 2 | CHL453 | T4 | WES | 76.88 |
| Patient 2 | CHL454 | T5 | WES | 131.36 |
| Patient 2 | CHL455 | T6 | WES | 116.52 |
| Patient 2 | CHL456 | T7 | WES | 87.2 |
| Patient 2 | CHL457 | T1 | WES | 85.63 |
| Patient 2 | WHL363 | RT1 | WGS | 31.80 |
| Patient 2 | WHL364 | RT2 | WGS | 36.77 |
| Patient 2 | WHL365 | RT3 | WGS | 34.84 |
| Patient 2 | WHL366 | RT4 | WGS | 36.35 |
| Patient 3 | CHL445 | N | WES | 77.36 |
| Patient 3 | CHL446 | T1 | WES | 71.38 |
| Patient 3 | CHL447 | T2 | WES | 76.48 |
| Patient 3 | CHL448 | T3 | WES | 71.61 |
| Patient 3 | CHL449 | T4 | WES | 64.54 |
| Patient 4 | WHL015 | T3 | WGS | 35.36 |
| Patient 4 | WHL016 | T1 | WGS | 35.7 |
| Patient 4 | WHL017 | N | WGS | 36.19 |
| Patient 4 | WHL018 | T2 | WGS | 35.82 |
| Patient 5 | WHL019 | T1 | WGS | 34.57 |
| Patient 5 | WHL020 | N | WGS | 35.63 |
| Patient 5 | WHL021 | T2 | WGS | 36.42 |
| Patient 6 | WHL246 | N | WGS | 42.16 |
| Patient 6 | WHL247 | T1 | WGS | 42.31 |

| | | | | |
|---|---|---|---|---|
| Patient 6 | WHL248 | T2 | WGS | 41.03 |
| Patient 6 | WHL249 | T3 | WGS | 41.8 |
| Patient 6 | WHL250 | T4 | WGS | 40.8 |
| Patient 6 | WHL251 | T5 | WGS | 41.4 |
| Patient 7 | WHL252 | N | WGS | 41.4 |
| Patient 7 | WHL253 | T1 | WGS | 41.0 |
| Patient 7 | WHL255 | T3 | WGS | 33.1 |
| Patient 7 | WHL256 | T4 | WGS | 39.5 |
| Patient 8 | WHL351 | N | WGS | 35.62 |
| Patient 8 | WHL352 | T1 | WGS | 38.92 |
| Patient 8 | WHL353 | T2 | WGS | 36.44 |
| Patient 8 | WHL354 | T3 | WGS | 35.64 |
| Patient 8 | WHL355 | T4 | WGS | 35.81 |
| Patient 8 | WHL356 | T5 | WGS | 37.39 |
| Patient 8 | WHL357 | T6 | WGS | 33.60 |
| Patient 9 | WHL358 | N | WGS | 33.56 |
| Patient 9 | WHL359 | T1 | WGS | 37.91 |
| Patient 9 | WHL360 | T2 | WGS | 37.29 |
| Patient 9 | WHL361 | T3 | WGS | 34.86 |
| Patient 9 | WHL362 | T4 | WGS | 31.82 |

Mixed sequencing technologies were used for this work partly due to technological advances. Patient 2/3 were sequenced earlier in the queue and later samples were sequenced using whole genome sequencing due to availability of newer sequencing machines and the possibility to find viral integrations with WGS.

**Supplementary Table 3: TP53 mutations in the six patients**

| Patient ID | Chromosome:position | Nuc change | AA change | # mutations in COSMIC |
|---|---|---|---|---|
| Patient 2 | 17:7578190 | A659G | Y220C | 229 |
| Patient 3 | 17:7577534 | G747C | R249S | 346 |
| Patient 4 | 17:7577505 | A776T | D259V | 17 |
| Patient 5 | 17:7578457 | G473T | R158L | 72 |
| Patient 6 | 17:7577534 | G747T | R249S | 346 |
| Patient 9 | 17:7578271 | A578G | H193R | 0 |

Based on transcript ID ENST00000269305

**Supplementary Table 4. Potential targetable mutations and their associated drugs**

| Patient | Gene/ Mutation | COSMIC | Domain | Drugs |
|---|---|---|---|---|
| 1 | KIT/F600Y | Yes[a] | No | Sorafenib/Regorafenib/ Pazopanib/ Ponatinib |
| 2 | EGFR/D916N | Yes[a] | No | Cetuximab/Erlotinib/Panitumumab |
| 2 | F2/D398Y | No | Yes | Tamoxifen |
| 3 | RET/ N962I | No | Yes | Sorafenib/Regorafenib/ Ponatinib/Cabozantinib |
| 6 | MAP2K2/A62S | No | No | Trametinib |
| 8 | PIK3CA/ E545K | Yes | Yes | Buparlisib/Alpelisib |
| 9 | ERBB4/C330X (stop gain) | No | Yes | Pertuzumab |

[a]: identical position to the COSMIC database, but with different mutation type.

**Supplementary Table 5. The HBV integrations found in three cases**

| PatientID | Tumor_sector | Chromosome | Position | # reads |
|---|---|---|---|---|
| Patient 1 | T8 | chr2 | 175017803 | 27 |
| Patient 1 | T11 | chr17 | 21215894 | 11 |
| Patient 1 | T0 | chr1 | 12637110 | 2 |
| Patient 1 | T6 | chr17 | 21215894 | 10 |
| Patient 1 | T5 | chr10 | 86930718 | 2 |
| Patient 1 | T1 | chr5 | 1296223 | 4 |
| Patient 1 | T9 | chr5 | 1295132 | 2 |
| Patient 1 | T10 | chr17 | 21215894 | 41 |
| Patient 1 | T2 | chr2 | 175017803 | 26 |
| Patient 1 | T8 | chr5 | 1295178 | 9 |
| Patient 1 | T9 | chr2 | 175017803 | 22 |
| Patient 1 | T7 | chr2 | 175017803 | 42 |
| Patient 1 | T11 | chr2 | 175017803 | 27 |
| Patient 1 | T6 | chr5 | 1295178 | 13 |
| Patient 1 | T4 | chr2 | 175017803 | 41 |
| Patient 1 | T3 | chr2 | 175017803 | 30 |
| Patient 1 | T10 | chr2 | 175017803 | 69 |
| Patient 1 | T12 | chrX | 125781079 | 2 |
| Patient 1 | T11 | chr5 | 1295178 | 10 |
| Patient 1 | T6 | chr2 | 175017756 | 14 |
| Patient 1 | T5 | chr17 | 21215894 | 5 |
| Patient 1 | T4 | chr5 | 1295178 | 6 |
| Patient 1 | T8 | chr17 | 21215894 | 15 |
| Patient 1 | T2 | chr17 | 21215894 | 16 |
| Patient 1 | T7 | chr17 | 21215894 | 14 |
| Patient 1 | T3 | chr17 | 21215894 | 14 |
| Patient 1 | T4 | chr17 | 21215894 | 17 |

| | | | | |
|---|---|---|---|---|
| Patient 1 | T2 | chr5 | 1295166 | 9 |
| Patient 1 | T5 | chr5 | 1295178 | 5 |
| Patient 1 | T7 | chr5 | 1295178 | 12 |
| Patient 1 | T3 | chr5 | 1295178 | 6 |
| Patient 1 | T5 | chr2 | 175017803 | 22 |
| Patient 1 | T10 | chr5 | 1295178 | 24 |
| Patient 1 | T1 | chr17 | 21215894 | 6 |
| Patient 1 | T1 | chr5 | 1295178 | 3 |
| Patient 1 | T3 | chr4 | 3076332 | 2 |
| Patient 1 | T9 | chr17 | 21215894 | 5 |
| Patient 1 | T9 | chr5 | 1295867 | 2 |
| Patient 1 | T1 | chr2 | 175017803 | 5 |
| Patient 1 | T9 | chr5 | 1296223 | 4 |
| Patient 1 | RT1 | Chr2 | 175017809 | 18 |
| Patient 1 | RT1 | Chr5 | 1295178 | 7 |
| Patient 1 | RT1 | Chr17 | 21215894 | 22 |
| Patient 1 | RT2 | Chr2 | 175017809 | 37 |
| Patient 1 | RT2 | Chr5 | 1295178 | 6 |
| Patient 1 | RT2 | Chr17 | 21215894 | 11 |
| Patient 6 | T4 | 12 | 60403370 | 3 |
| Patient 6 | T5 | 17 | 18704095 | 44 |
| Patient 6 | T5 | 2 | 58491341 | 23 |
| Patient 6 | T1 | 3 | 133210434 | 19 |
| Patient 6 | T4 | 3 | 133210434 | 26 |
| Patient 6 | T3 | 13 | 53779493 | 18 |
| Patient 6 | T1 | 12 | 7874214 | 3 |
| Patient 6 | T4 | 17 | 18704095 | 43 |
| Patient 6 | T5 | 3 | 133210434 | 19 |
| Patient 6 | T5 | 13 | 53779061 | 22 |

| | | | | |
|---|---|---|---|---|
| Patient 6 | T5 | 13 | 53779493 | 43 |
| Patient 6 | T2 | 11 | 67749363 | 21 |
| Patient 6 | T1 | 2 | 58353638 | 2 |
| Patient 6 | T3 | 11 | 67749363 | 18 |
| Patient 6 | T1 | 13 | 53779493 | 41 |
| Patient 6 | T2 | 17 | 18704095 | 56 |
| Patient 6 | T2 | 8 | 53315531 | 12 |
| Patient 6 | T5 | 8 | 53315531 | 19 |
| Patient 6 | T5 | 2 | 58353638 | 2 |
| Patient 6 | T4 | 13 | 53779493 | 44 |
| Patient 6 | T3 | 3 | 133210434 | 21 |
| Patient 6 | N | 1 | 84322973 | 2 |
| Patient 6 | T4 | 3 | 133210999 | 6 |
| Patient 6 | T2 | 3 | 133210434 | 12 |
| Patient 6 | T5 | 6 | 166679498 | 2 |
| Patient 6 | T2 | 10 | 42380255 | 2 |
| Patient 6 | T4 | 11 | 67749363 | 21 |
| Patient 6 | T1 | 17 | 18704095 | 49 |
| Patient 6 | T1 | 8 | 53315531 | 16 |
| Patient 6 | T3 | 8 | 53315531 | 25 |
| Patient 6 | T1 | 11 | 67749363 | 28 |
| Patient 6 | T1 | 2 | 58491341 | 25 |
| Patient 6 | T2 | 2 | 58491341 | 17 |
| Patient 6 | T4 | 8 | 53315531 | 16 |
| Patient 6 | T2 | 2 | 58353638 | 2 |
| Patient 6 | T5 | 17 | 19874990 | 2 |
| Patient 6 | T4 | 17 | 19874990 | 10 |
| Patient 6 | T5 | 11 | 67749363 | 15 |
| Patient 6 | T3 | 2 | 58353638 | 2 |

| | | | | |
|---|---|---|---|---|
| Patient 6 | T4 | 2 | 89875342 | 2 |
| Patient 6 | T2 | 17 | 19874990 | 2 |
| Patient 6 | T4 | 2 | 58353638 | 2 |
| Patient 6 | T2 | 13 | 53779493 | 35 |
| Patient 6 | T1 | 17 | 19874990 | 2 |
| Patient 6 | T3 | 17 | 18704095 | 59 |
| Patient 6 | T3 | 17 | 19874990 | 2 |
| Patient8 | T5 | chr10 | 55471926 | 29 |
| Patient8 | T6 | chr11 | 69454575 | 34 |
| Patient8 | T4 | chr10 | 55451328 | 5 |
| Patient8 | T4 | chr11 | 69454509 | 15 |
| Patient8 | T5 | chr5 | 1295362 | 13 |
| Patient8 | T3 | chr19 | 42749633 | 5 |
| Patient8 | N | chr1 | 93475296 | 6 |
| Patient8 | T1 | chr10 | 55451328 | 5 |
| Patient8 | T3 | chr5 | 1295362 | 13 |
| Patient8 | T5 | chr10 | 55451328 | 32 |
| Patient8 | T3 | chr11 | 69454575 | 42 |
| Patient8 | T5 | chr19 | 42749633 | 5 |
| Patient8 | T6 | chr5 | 1295362 | 10 |
| Patient8 | T3 | chr19 | 42749425 | 3 |
| Patient8 | T4 | chr5 | 1295362 | 10 |
| Patient8 | T2 | chr5 | 1295577 | 14 |
| Patient8 | T5 | chr19 | 42749698 | 5 |
| Patient8 | N | chr19 | 42721466 | 2 |
| Patient8 | T6 | chr10 | 55471926 | 5 |
| Patient8 | T1 | chr11 | 69454575 | 36 |
| Patient8 | T4 | chr19 | 42749698 | 5 |
| Patient8 | T6 | chr19 | 42749633 | 5 |

| | | | | |
|---|---|---|---|---|
| Patient8 | T5 | chr11 | 69454575 | 45 |
| Patient8 | T6 | chr10 | 55451328 | 5 |
| Patient8 | T3 | chr10 | 55471926 | 5 |
| Patient8 | T2 | chr10 | 55471926 | 5 |
| Patient8 | T4 | chr19 | 42749633 | 5 |
| Patient8 | T4 | chr10 | 55471926 | 5 |
| Patient8 | T2 | chr19 | 42749633 | 5 |
| Patient8 | T1 | chr19 | 42749633 | 5 |
| Patient8 | T1 | chr10 | 55471926 | 5 |
| Patient8 | T6 | chr2 | 75698946 | 2 |
| Patient8 | T2 | chr11 | 69454575 | 25 |
| Patient8 | T4 | chr11 | 69454575 | 9 |
| Patient8 | T3 | chr19 | 42749698 | 5 |
| Patient8 | T6 | chr19 | 42749698 | 5 |
| Patient8 | T3 | chr10 | 55451328 | 5 |
| Patient8 | T2 | chr5 | 1295371 | 5 |
| Patient8 | T2 | chr10 | 55451328 | 5 |
| Patient8 | T2 | chr19 | 42749698 | 5 |
| Patient8 | T1 | chr5 | 1295362 | 10 |
| Patient8 | T5 | chr2 | 160515153 | 2 |
| Patient8 | T1 | chr19 | 42749698 | 5 |

**Supplementary Table 6. Viral load across the four HBV positive cases**

| Patient | Viral dosage |
|---|---|
| 1 (WGS) | 108,656,187 copies/ml (8.04 LOG) |
| 3 (Exome) | NA |
| 6 (WGS) | 24,822,463 copies/ml (7.39 LOG) |
| 7 (WGS) | 797 copies/ml (2.90 LOG) |
| 8 (WGS) | 87,510 copies/ml (4.94 LOG) |

# Supplementary Notes

## Supplementary Note 1. Experimental validation of somatic variants

Somatic variation calling has been well explored and calibrated in the field. In our somatic variant calling, we used Mutect to call somatic changes across the WGS/WES. Using sequenom platform (a Mass Spectrometry based technology) [1], we validated 30 somatic variants across all 12 samples (11 T and 1N) from the first patient. We found that, the validation rate of somatic variants is 96% (27 out 28 sites are successfully validated as the somatic variants. 2 of the sites are potential errors due to sequenom and they showed positive allele frequency in the control/hapmap sample). It is important to emphasize that the sequenom can sometimes have some baseline read out in a few cases due to technical issues.

## Supplementary Note 2. Mutational burden and comparison to other tumor types

From public domains, we have downloaded a large number of WES/WGS datasets for HCC (Methods). The mutation information for other tumors was downloaded from http://www.tumorportal.org. When plotting the mutational profile against many other tumor types, we found that HCC is similar to the colorectal cancer and the overall mutation rate is intermediate comparing to many other cancer types. The somatic mutation frequency is also curated for all each gene across the HCC (Shown in Figure 1b).

## Supplementary Note 3. Phylogenetic reconstruction and Fst calculation

Using a statistical approach known as the bootstrapping[2], we assessed the statistical confidence in the phylogenetic relationship across all the patients. We found that, the phylogenetic tree is highly consistent (bootstrap confidence >0.7) for patients with enough of genetic variability (Supplementary Fig. 5, except patient 1 and 4). Patient 1 and 4, which the tumor sectors are highly similar, the statistical evidence for the phylogenetic relationships is less strong.

A related problem with constructing the phylogenetic relationship is that whether we can combine multiple genetic changes (e.g. CNV together with SNV/Indel) in inferring the evolutionary trajectory. In order to explore this approach, we first calculated the genome wide copy number profiles (logR and BAF) using an ASCAT like procedure (https://github.com/cancerit/ascatNgs) for all tumor sectors independently. We then used a computational procedure based on penalized least squares minimization (Piecewise Constant Fits)[3]. This allowed us to jointly segment the copy number profiles across all sectors for each patient. Subsequently, we tabulated copy number variations across patient sectors taking into account the purity values of the sectors (slightly different cutoffs for each sectors depending on the tumor purity). With this binary presence and absence data, we combined mutation data (SNV and indel) together with the CNV information to build the phylogenetic tree. We found that, adding the CNV profiles are not changing the phylogeny inferred solely from the mutation data (Data not shown). This is because the number of CNV events is relatively small (often less than 100) and most of them are truncal.

In addition to the phylogenetic inference using WGS sequencing, we also inferred phylogenetic relationships using exome part of the whole genome. In Supplemenary Fig. 5b, we showed all the phylogenetic trees based on WES and most of the phylogenetic trees are identical to the whole genome trees. The only exception is patient 1 and 8. Patient 1's phylogenetic relationship cannot be definitively resolved due to very recent divergence (Fig.2). The two trees for patient 8 are only slightly different. Statistical fluctuations or other factors (including natural selection in exome regions) can potentially contribute to the slight difference in tree topology.

FST was first developed by Sewall Wright[4]. Wright wanted to model population structure in breeds of livestock and in natural populations. Following Wright's seminal work, subsequent developments by Masatoshi Nei[5], C. Clark Cockerham[6] and Bruce Weir[7] have brought FST to an analysis of variance (ANOVA) framework. FST has been interpreted as the proportion of the total variance in allele frequency caused

by allele frequency differences between populations. Under this framework, we believe (as did others as referenced) that FST can be calculated without references to the underlying biological model and thus are applicable to cancer cell populations.

Since the variant frequency is globally affected by tumor purity, in order to calculate the population differentiation between samples, we need to take into account this factor. Let's denote a set of sectors ($T_1$, $T_2$… $T_N$ with estimated purity $p_1$, $p_2$… $p_N$). Let's denote the maximum purity of all the sectors to be $p_{max}$. For all tumor sectors, we want to adjust the purity to be the same purity (e.g. $p_{max}$). For a given SNV with x mutant reads, n-x wild type reads, we resample n reads with mutant allele having a higher probability than the wild type allele. This is done by having mutant allele being $p_{max}/p_i$ more likely to be sampled than the wild type allele. We do this for all SNVs across the genome for all sectors. Fst values were then calculated based on the resampled read counts.

**Supplementary Note 4. Computational modeling of the tumor populations.**

Three dimensional modeling of tumor populations have been conducted in many earlier studies[8,9]. The intention here is to test whether the IBD and spatial segregating can be generated using simple computational models. Other than the simple spatial model, we also added extra parameter combinations: 1) different levels of migration, 2) different levels of growth rate, 3) differential growth rate driven by adaptive evolution (e.g. a proportion of sites are adaptive). We found that, under a wide variety of models, the isolation by distance relationships (e.g. Fig. 3b) can be easily simulated (data not shown). In other words, very simple models can generate the pattern we observed in HCC.

**Supplementary Note 5. HBV integration**

From all output of the program, we first collapsed integration sites whose distances are less than 50bp in a single sample. For patient 1, this left us with 73 candidate integration sites across all samples (tumor and normal). From this set, all integration

sites whose supporting read is less than 2 were filtered away. We ended up with 45 high fidelity integration sites (Supplementary Table S5). For patient 6, we identified 47 high fidelity integration sites in 6 samples (5 tumors and 1 normal) (Supplementary Table S5). For patient 8, 43 high fidelity integration sites are found across 7 samples (6 tumors and 1 normal). Integration sites whose distances in different samples are less than 2kb were treated as the same integration site. This left us with 6 unique integration sites for patient 1 and 15 unique integrations for patient 6 and 9 unique integrations for patient 8 (Fig. 3b).

Using publically curated 1027 integration sites (Methods). A slide window procedure (window size is 20kb, step size is 10kb) is implemented across the genome. A few candidate regions (24 windows with >3 integrations) were identified as integration hotspots (for example, TERT, MLL4 etc). There are three hotspot integrations found in the three cases. That's TERT integration found on chromosome 5 for patient 1 and 8 and a noncoding integration found on chromosome 10 (Supplementary Figure 8) in patient 6.

## Supplementary References


1 Gabriel, S., Ziaugra, L. & Tabbaa, D. SNP genotyping using the Sequenom MassARRAY iPLEX platform. *Current protocols in human genetics / editorial board, Jonathan L. Haines ... [et al.]* **Chapter 2**, Unit 2 12, doi:10.1002/0471142905.hg0212s60 (2009).

2 Felsenstein, J. Confidence limits on phylogenies: an approach using the bootstrap. *Evolution*, 783-791 (1985).

3 Nilsen, G. *et al.* Copynumber: Efficient algorithms for single-and multi-track copy number segmentation. *BMC genomics* **13**, 591 (2012).

4 Wright, S. The genetical structure of populations. *Annals of eugenics* **15**, 323-354 (1951).

5 Nei, M. F-statistics and analysis of gene diversity in subdivided populations. *Annals of human genetics* **41**, 225-233 (1977).

6 Cockerham, C. C. Analyses of gene frequencies. *Genetics* **74**, 679-700 (1973).

7 Weir, B. S. & Cockerham, C. C. Estimating F-statistics for the analysis of population structure. *evolution*, 1358-1370 (1984).

8 Sottoriva, A. *et al.* A Big Bang model of human colorectal tumor growth. *Nature genetics* **47**, 209-216 (2015).

9 Waclaw, B. *et al.* A spatial model predicts that dispersal and cell turnover limit intratumour heterogeneity. *Nature*, doi:10.1038/nature14971 (2015).
